# Real‐time motion tracking of a gold implant in water by Cherenkov lights

**DOI:** 10.1002/acm2.70684

**Published:** 2026-07-07

**Authors:** Keita Okazaki, Eric Brost, Gopishankar Natanasabapathi, Yoichi Watanabe

**Affiliations:** ^1^ Department of Radiation Oncology University of Minnesota Minneapolis Minnesota USA; ^2^ Department of Radiation Oncology Thomas Jeffesion University Philadelphia Pennsylvania USA; ^3^ Department of Radiation Oncology Mayo Clinic Rochester Minnesota USA; ^4^ Department of Radiation Oncology Dr. B. R. A. Institute Rotary Cancer Hospital All India Institute of Medical Sciences New Delhi Delhi India

**Keywords:** Cherenkov light, gold implant, Monte Carlo, Motion‐tracking, radiotherapy

## Abstract

**Background:**

The intra‐fraction motion of tumors during radiation therapy can be monitored using imaging systems based on X‐ray imaging (e.g., kV/MV radiography or CBCT) or magnetic resonance imaging (MRI). However, these techniques have limitations, including additional ionizing radiation dose and the need for complex, expensive equipment. Hence, a non‐invasive, lower‐cost motion‐tracking approach that does not add radiation would be beneficial for routine clinical applications.

**Purpose:**

To study the feasibility of tracking the dynamic motion of a small gold marker in water by using Cherenkov photons produced by MV photon beams, through Monte Carlo simulations and experiments.

**Methods:**

The GAMOS Monte Carlo code, which includes an optical simulation module, was used to calculate the deposited dose and Cherenkov‐light yield in a water phantom. Phase‐space files for 6, 10, 15, and 10 MV flattening filter‐free (FFF) beams served as photon sources. The simulation setup consisted of a 20 × 20 × 20 cm^3^ water phantom with an 8 × 12 × 0.25 mm^3^ gold planar implant at its center. Percent depth dose (PDD) and Cherenkov‐light yield were evaluated throughout the phantom and near the gold implant.

For experimental validation, a 20 × 20 × 20 cm^3^ glass water tank was placed on a programmable platform executing sinusoidal, sawtooth, and sharkfin motions (± 20 mm amplitude; 10‐ and 60‐s cycles). A 5 × 5 cm^2^ 10 MV FFF beam (2400 MU/min) irradiated the gold implant. A scientific CMOS camera (f/2.0) positioned at 80 cm from the implant captured Cherenkov images. Repeatability was quantified by the standard deviation (SD) across motion cycles. The accuracy was assessed as the absolute difference between the bright‐room reference position and the Cherenkov‐derived position. Images of the implant in sinusoidal motion were also acquired during delivery of a two‐arc brain volumetric modulated arc therapy (VMAT) plan (Arc A and Arc B).

**Results:**

Monte Carlo simulations showed that Cherenkov light yield throughout the phantom closely correlated with the PDD for all beam energies. The Cherenkov light yield upstream of the gold implant increased by 6.12%, 7.16%, 9.11%, and 7.01% compared to the Cherenkov light yield without the gold implant for 6, 10 FFF, 10, and 15 MV beams, respectively.

Experiments confirmed that the beam‐facing side of the gold implant appeared brighter than the opposite side, indicating enhanced backscattered electron production. Cherenkov‐based positional measurements of the periodically moving implant showed close agreement with bright‐room references. For static beams, accuracy across motion patterns was < 0.70 mm with repeatability < 0.2 mm. For the VMAT plan, the accuracies were < 0.50 mm (Arc A) and < 0.70 mm (Arc B).

**Conclusion:**

The Cherenkov‐light yield was strongly correlated with the deposited dose and showed a notable enhancement around the gold implant. Cherenkov light imaging successfully tracked the dynamic motion of a gold implant in water under high‐dose rate conditions, demonstrating its potential for real‐time fiducial tracking in radiation therapy without additional radiation exposure.

## 1 INTRODUCTION

External beam radiation therapy (EBRT) is utilized to treat cancer patients. During EBRT, Cherenkov light has been observed. It is a phenomenon that arises when photon beams induce ionization in a medium, leading to photon emission by secondary electrons.^1^, ^2^, ^3^ Notably, Cherenkov light is produced when charged particles traverse a medium at speeds exceeding the phase velocity of light in that medium.^4^ This unique property of Cherenkov light has driven research into dosimetry applications, such as quality assurance in radiation therapy.^5^, ^6^, ^7^, ^8^ Previous studies have demonstrated that the intensity of Cherenkov light generated by radiation treatment beams is directly proportional to the dose deposited.^5^, ^9^ Cherenkov imaging has been successfully applied during total skin electron irradiation (TSEI), where acquired Cherenkov images demonstrated that light intensity was proportional to.^10^ In addition, the use of near‐infrared (NIR) light has attracted attention for imaging through tissue, as NIR photons experience significantly less absorption and scattering than visible wavelengths, thereby improving penetration depth and signal recovery for in vivo applications.^11^, ^12^, ^13^, ^14^ These findings support the potential of the Cherenkov imaging technique, especially NIR‐based optical imaging, to enhance treatment monitoring and target localization in radiation therapy.^15^

A tiny gold marker implant is often used to identify the tumor location for EBRT. This technique is used to accurately direct radiation beams toward the target, minimizing exposure of surrounding healthy tissues. Image‐Guided Radiation Therapy (IGRT) usually achieves target and critical structure localization by taking images with MV or kV X‐rays immediately before or during treatment, at the expense of additional patient doses and increased image acquisition time.

Recently, linear accelerators incorporating magnetic resonance imaging (MRI), known as MR‐Linacs, have been introduced to enable real‐time tumor and surrounding organ localization during irradiation in clinical settings.^16^, ^17^, ^18^ Unlike cone‐beam computed tomography (CBCT), MRI does not cause additional ionizing radiation exposure. Hence, the MRI‐linac reduces the overall imaging dose burden on patients, making it an attractive modality for IGRT, particularly when repeated imaging is necessary to ensure treatment precision. However, patients with certain metallic implants, such as non‐MR‐compatible pacemakers, implantable cardioverter defibrillators (ICDs), or ferromagnetic surgical clips, are generally contraindicated for MR‐Linac treatment due to safety concerns and the potential for severe image artifacts. In this regard, Cherenkov imaging offers a unique advantage by enabling real‐time visualization of the delivered radiation field without any additional radiation dose.

In this study, we investigated the use of Cherenkov light to localize a gold implant marker in water during irradiation, enabling dynamic, real‐time fiducial tracking as a surrogate for target motion. It is known that the presence of a high atomic number material, such as gold, in a medium enhances the dose around the implant due to increased electron production,^19^ resulting in increased Cherenkov light emission around the gold implant. Hence, the Cherenkov lights can be used to visualize the marker more effectively. Furthermore, since the visualization includes both static and moving objects, we can use the Cherenkov imaging technique to track the gold implant marker in real time.

We first conducted Monte Carlo simulations to demonstrate the feasibility of the proposed technique. We investigated variations in the dose and Cherenkov light yield near a gold implant for photon beams with various energies. Next, the idea was validated through experiments in which a scientific‐grade CMOS camera captured video images of a moving gold implant placed in a water phantom on a moving platform. To quantify the accuracy of position localization, we analyzed the discrepancies between the captured images and the expected trajectory of the gold implant.

## 2 MATERIALS AND METHODS

### 2.1 Monte Carlo simulations

The GAMOS Monte Carlo code is based on GEANT4 and provides an easy‐to‐use, flexible framework. This study utilized GAMOS to calculate two physical quantities: deposited dose and Cherenkov light yield in water.^20^

Simulations were performed for Varian TrueBeam (Varian, a Siemens Healthineers Company, Palo Alto, CA, USA) photon beams at energies of 6 MV, 10 MV, 15 MV, and 10 MV flattening filter‐free (FFF). The photon‐source data were obtained from phase‐pace files provided by Varian.^21^ The original phase‐space files created by the vendor were positioned 26.7 cm downstream of the target, located below the monitor ionization chamber but upstream of the primary collimator jaws, which were configured to define a 5 × 5 cm^2^ field size. To improve computational efficiency and reduce unnecessary particle tracking in the upstream head components, we generated a new phase‐space file for each photon beam. These files were scored at a plane located 50 cm below the target, corresponding to 6.25 cm downstream of the collimator jaws. This new phase‐space records the particle characteristics after the beam has passed through the jaws, thereby reducing the simulation geometry and focusing computational resources on the region of interest within the phantom.

The simulation geometry consisted of a 20 × 20 × 20 cm^3^ cubic water phantom, with a source‐to‐surface distance (SSD) of 100 cm. An 8 × 12 mm^2^ area of a 0.25 mm‐thick gold plate was placed at a 10 cm depth at the center of the phantom. The entire water phantom was discretized into rectilinear voxels of 10 × 10 × 5 mm^3^, and the deposited dose was scored in every voxel. To generate the percent depth‐dose (PDD) curves, we used a 20 × 20 × 5 mm^3^ volume, centered on the beam axis and spaced at 5‐mm depth intervals. The PDD dose was calculated as an average of the doses scored by four 10 × 10 × 5 mm^3^ rectilinear voxels.

**FIGURE 1:**
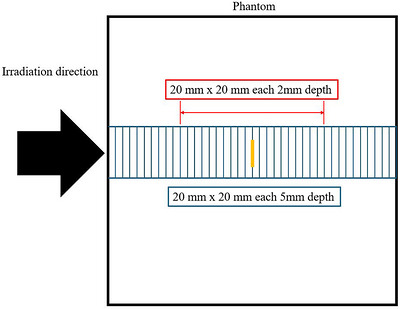
A cubic water phantom and scoring regions in the GAMOS simulation. The blue boxes within the phantom represent scoring regions for dose calculation, each measuring 20 mm × 20 mm × 5 mm. The red‐colored region extends ± 5 cm from the gold plate and contains 50 scoring volumes to evaluate Cherenkov light yield in the vicinity of the gold. In this region, scoring was performed at 2 mm depth intervals.

Figure 1 depicts the scoring region used for Cherenkov photon detection, which was restricted to the beam‐axis region to improve statistical reliability while maintaining geometric consistency with the dose scoring. For Cherenkov light yield, the scoring volumes were identical to those used for deposited dose calculations. Additionally, higher‐resolution calculations were performed within 20 × 20 × 2 mm^3^ volumes at 2‐mm depth intervals along the central axis around the gold plate, spanning from + 5 cm (upstream) to −5 cm (downstream) relative to the gold implant, to capture the localized enhancement of Cherenkov photon emission. The Cherenkov light yield was defined as the total number of Cherenkov optical photons generated within the scoring volume for each depth bin, normalized by the number of particles incident on the phantom (i.e., particles crossing the phantom entrance plane downstream of the jaws). This normalization was used to compare the relative Cherenkov production efficiency in the phantom across beam energies under Monte Carlo simulations. Because both the deposited dose and Cherenkov light yield were scored within the same volumes and normalized by the same number of histories, this approach does not introduce bias in comparisons between beams with different fluence profiles (e.g., flattened vs. flattening‐filter‐free beams).

The maximum number of particle records available in the phase‐space files varied by beam energy: 92,158,350 for 6 MV, 97,545,816 for 10 MV, 90,902,962 for 10 MV FFF, and 95,137,030 for 15 MV, respectively. These counts represent the total number of particles stored in the phase‐space files, including photons, electrons, and positrons. The simulations were performed multiple times, using the maximum number of events available in the original phase‐space files, by altering the random seed with the /gamos/random/setSeeds command of GAMOS. This approach ensured that the statistical uncertainty in both the deposited‐dose and Cherenkov‐light‐yield calculations was reduced to below 1%.

### 2.2 Experiment

**FIGURE 2:**
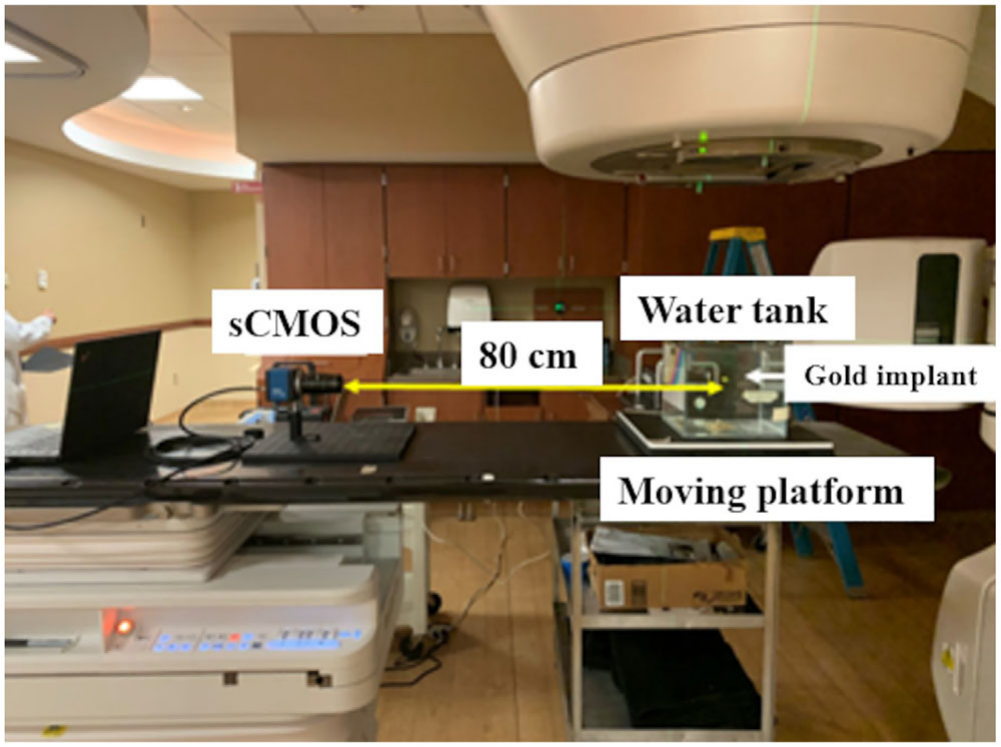
Experimental setup.

Experiments were designed to assess the feasibility of tracking marker motion for both static and dynamic beams. For the latter experiment, the beam intensity, the beam angle, and the position of the gold implant were varied simultaneously. The static and dynamic beams utilized for the experiments were a 10 MV FFF photon beam from a Varian TrueBeam. The irradiation field size of the static beam was 5 × 5 cm^2^ at a 90‐degree gantry angle. The dose rate was set to 2400 MU/min for the static and dynamic beams. The experimental setup is shown in Figure 2. A glass container of 20 × 20 × 20 cm^3^ filled with water was placed on a moving platform (Dynamic Platform Model 008PL, Sun Nuclear, Melbourne, FL, USA), and a thin planar gold implant of 8 mm x 12 mm with 0.25 mm thickness was placed at the center of the container at 9.9 cm depth. According to the manufacturer's specifications, the motion platform has a motion accuracy of ± 0.1 mm. A scientific CMOS camera (pco.panda 4.2, PCO‐TECH Inc., Romulus, MI, USA) was positioned 80 cm from the gold marker, orthogonal to the beam central axis in the horizontal plane. The camera's f‐number was 2.0. To facilitate marker detection, the phantom was rotated 20 degrees toward the gantry.

For the static beam experiments, the platform was programmed to generate three motion patterns—sinusoidal, sawtooth, and sharkfin—with amplitudes ranging from −20 mm to +20 mm and cycle durations of either 10 or 60 s. The platform moved along the beam direction. For the 10‐s cycles, both the exposure time and image acquisition interval were set to 100 ms. For the 60‐s cycles, the exposure time remained 100 ms, but images were acquired at 1‐s intervals. Image acquisition was performed under both bright and dark room conditions for at least two complete motion cycles. The bright‐room data enabled visualization of the gold implant's translational motion, whereas the dark‐room data captured Cherenkov‐based motion detection of the gold implant. The bright‐room motion data was used as the reference, against which the position measured in the dark room was compared to quantify accuracy. Repeatability was quantified as the difference across motion cycles at matched time points by treating the two cycles as independent measurements. It is defined with the data of two cycles as:

$$\begin{array}{c} Repeatability=\frac{\left|y_{1}\left(j\right)-y_{2}\left(j\right)\right|}{\sqrt{2}}\;\left(1\right) \end{array}$$

where $y_{1}\left(j\right)$ denotes the position of the first cycle at time *j*, and $y_{2}\left(j\right)$ denotes the position of the second cycle at the time *j*.

The positional error was defined as the absolute difference between the bright‐room reference position and the Cherenkov‐derived position at matched time points. Then, accuracy was assessed as follows:

$$\begin{array}{c} Accuracy=\left|{\bar{y}}_{ref}\left(j\right)-{\bar{y}}_{Cherenkov}\left(j\right)\right|\;\left(2\right) \end{array}$$

where ${\bar{y}}_{ref}\left(j\right)$ is the mean position of the first and second cycle measured in a bright room at time *j*, and ${\bar{y}}_{Cherenkov}\left(j\right)$ is the mean position of the first and second cycle Cherenkov‐derived position at time *j*. Although the pixel size was 0.1 mm/pixel, peak localization was performed using Gaussian fitting to the intensity profile, and the fitted peak center provides a continuous (sub‐pixel) position estimate. The reported decimal places therefore reflect the numerical precision of the estimator rather than the intrinsic spatial sampling of the camera. The practical localization uncertainty, which is influenced by image SNR/blur and fitting variability, was evaluated empirically using the repeatability across repeated motion cycles and the bright‐room–referenced accuracy metrics.

For the dynamic beam experiment, a volumetric modulated arc therapy (VMAT) plan designed for brain cancer treatment was delivered. Two arcs were delivered: Arc A involved gantry rotation from 178° to 58° with a total of 537 MU, while Arc B ranged from 179° to 59°, delivering 778 MU. The platform was programmed to follow a sinusoidal motion profile. Image acquisition was conducted once in a completely dark room with a 1‐s exposure and acquisition interval (i.e., 60 frames per minute), independent of gantry speed. The gold implant moved laterally (left‐to‐right direction) in accordance with the standard linac coordinate system.

**FIGURE 3:**
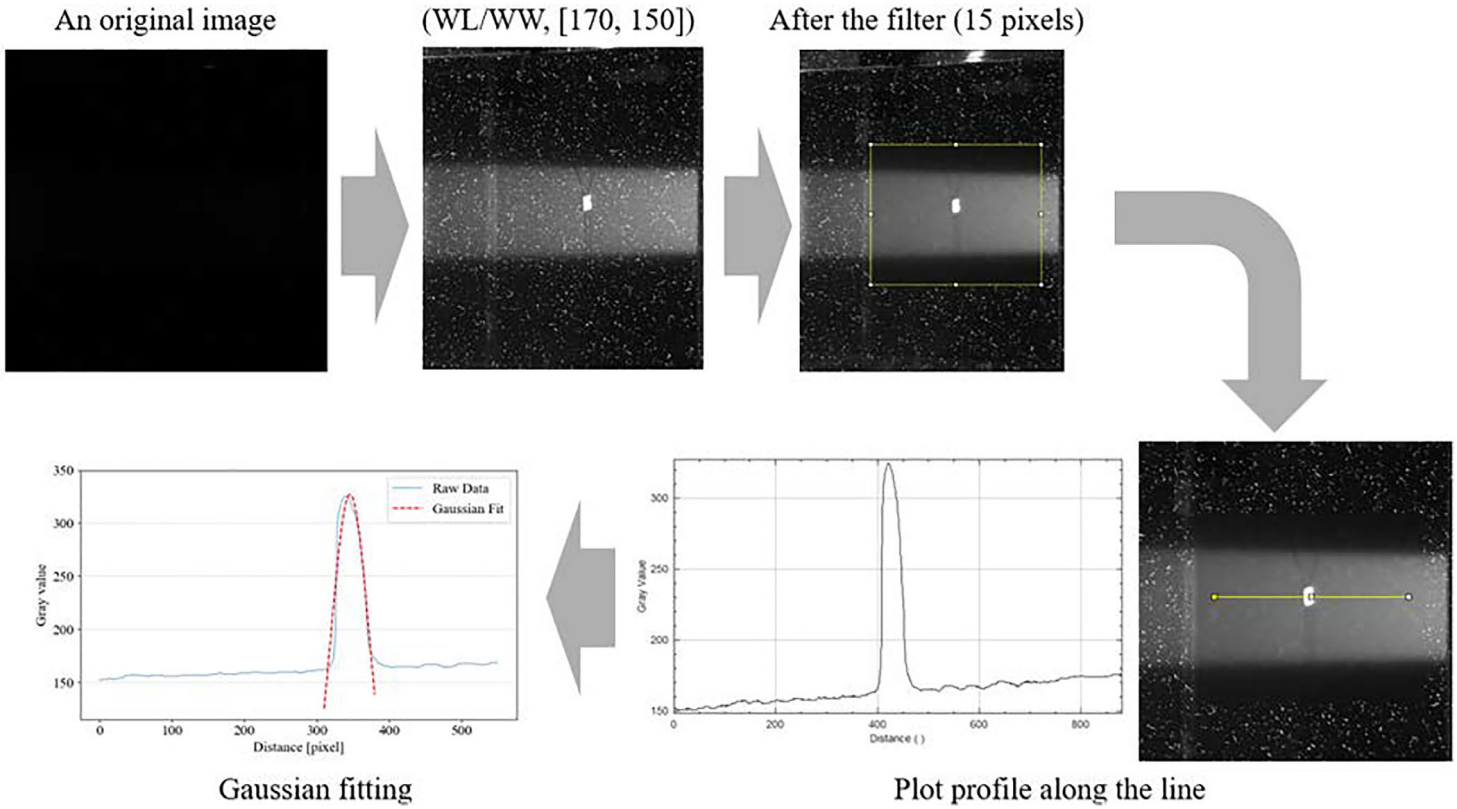
Analysis procedure of acquired Cherenkov images using ImageJ.

All acquired images were analyzed using ImageJ (NIH, Bethesda, MD, USA) according to the workflow illustrated in Figure 3.^22^ The pixel resolution was 0.1 mm. Because Cherenkov images exhibited an inherently low signal‐to‐noise ratio, the raw frames appeared predominantly dark. Therefore, a fixed window level/window width (WL/WW = 170/150) was applied to all frames to rescale intensities and improve marker visibility before the profile plot. This operation corresponds to a monotonic intensity remapping and was used to facilitate robust peak identification under low‐signal conditions. A median filter (15‐pixel kernel) was then applied to suppress noise; this step introduced no measurable shift in peak location within the image resolution. Next, a one‐dimensional intensity profile was extracted along the beam axis, and a Gaussian function was fitted to the intensity peak. The estimated gold‐implant position was defined as the center of the fitted Gaussian, corresponding to the midpoint of its full width at half maximum (FWHM).

## 3 RESULTS

### 3.1 Monte Carlo simulations

**FIGURE 4:**
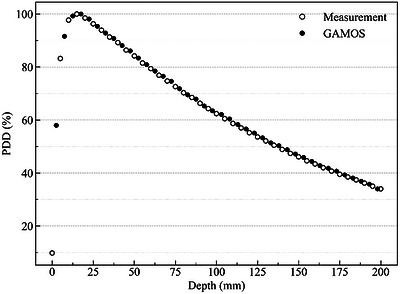
Percent depth dose (PDD) comparison using 6 MV photon beam between measurements and simulation in GAMOS.

The PDDs of all photon beams were calculated based on the deposited dose within the scored voxels. Figure 4 compares the GAMOS‐simulated PDD with the beam commissioning data for a 6 MV photon beam. The figure confirms the validity of the Monte Carlo simulations, including the geometric and radiation source models.

**FIGURE 5:**
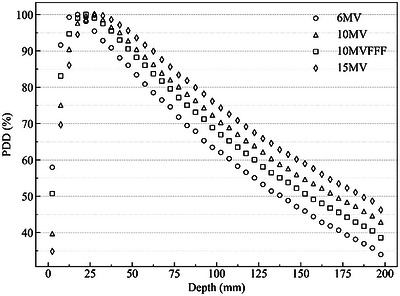
PDD distributions for the Varian photon beams. A source to surface distance was 100 cm. The scoring interval in the phantom was set to 5 mm in depth. The maximum dose for each beam was normalized to 100%.

Figure 5 shows the PDDs of 6, 10, 15, and 10 MV‐FFF photon beams. The statistical uncertainty of the PDD in the scored voxels was less than 1%. Among the energies tested, the 15 MV beam showed the highest PDD at a depth of 10 cm, due to its higher photon energy. In contrast, the 6 MV beam had the lowest PDD at the same depth. The 10 MV FFF beam contained more lower‐energy photons than the standard 10 MV beam, resulting in a slightly lower PDD than the conventional 10 MV beam.

**FIGURE 6:**
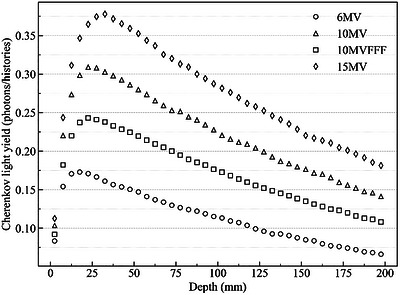
Cherenkov light yield distributions for the Varian photon beams. The Cherenkov light yield was defined as the number of Cherenkov photons generated within the scoring volume, divided by the number of particle histories.

The Cherenkov light yield, measured at 5 mm depth intervals and normalized to the number of particle histories, is shown in Figure 6. The statistical uncertainty of the Cherenkov light yield calculations in the scored volumes was less than 1%. The 15 MV beam produced the highest Cherenkov light yield, while the 6 MV beam had the lowest. The Cherenkov light yield for the 10 MV and 10 MV FFF beams was similar; however, due to the lower average photon energy of the 10 MV FFF beam, its Cherenkov light yield was slightly lower than that of the 10 MV beam.

**FIGURE 7:**
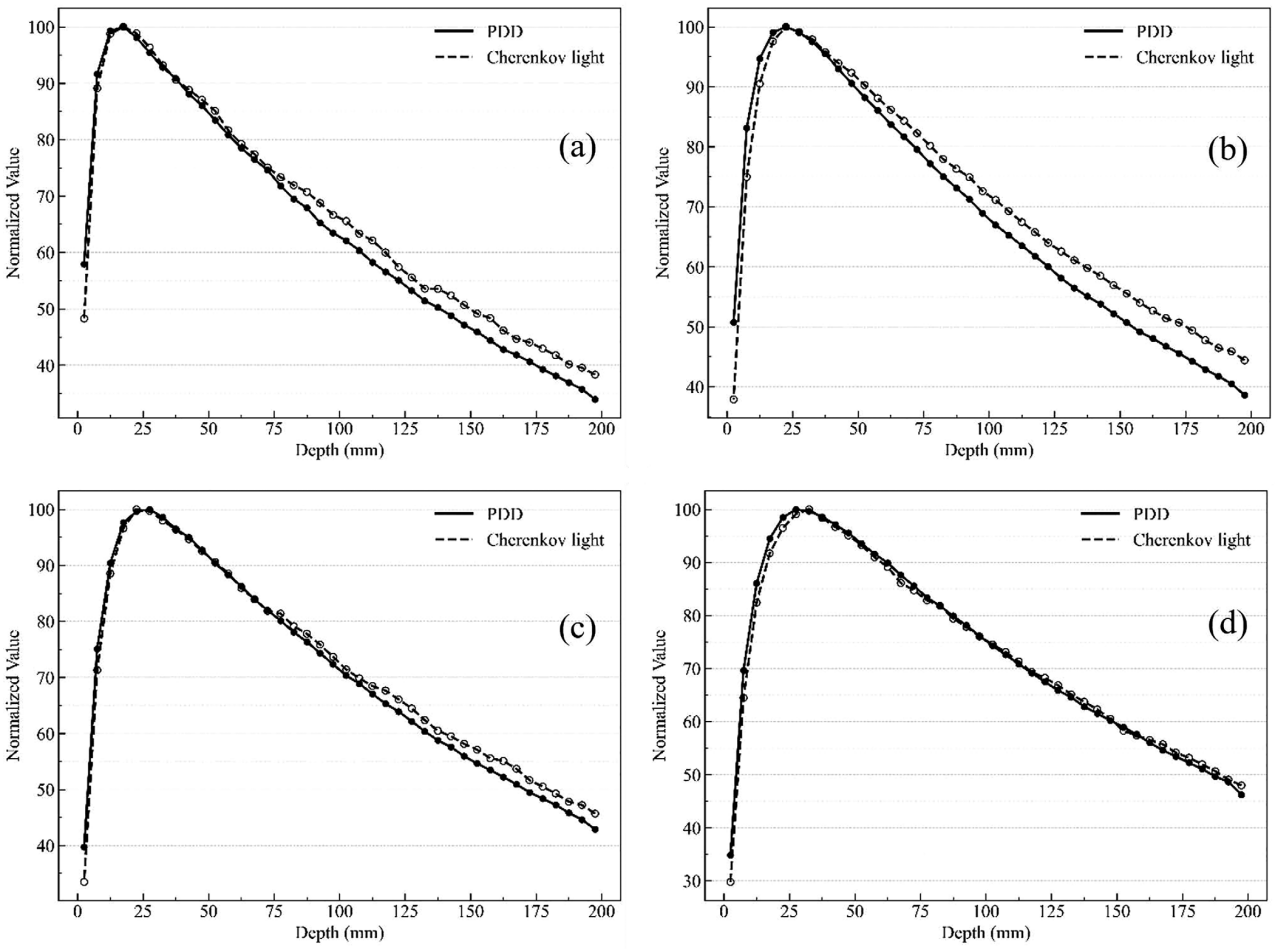
Comparison between PDD and Cherenkov light yield calculated in GAMOS. Panels (a), (b), (c), and (d) correspond to the 6 MV, 10 MV FFF, 10 MV, and 15 MV photon beams, respectively. The PDD was normalized using the same method described in Figure 5. The Cherenkov light yield was normalized such that the maximum value shown in Figure 6 corresponds to 100.

A comparison of the normalized PDD and Cherenkov light yield for all photon beams is shown in Figure 7. The normalized PDD was calculated using the same method as described in Figure 5. The normalized Cherenkov light yield was scaled so that the maximum value in Figure 6 equals 100. The Cherenkov light yield matched closely with the PDD near the depth of dose maximum (dmax) for all photon beams. The relative differences averaged over the entire depth (0 to 200 mm) were 5.0%, 6.7%, 2.9%, and 1.6% for 6, 10 FFF, 10, and 15 MV beams, respectively. Beyond a depth of 12.5 cm, the PDD for the 6 MV and 10 MV FFF beams declined more rapidly than the corresponding Cherenkov light yield.

The Cherenkov light yield at a depth of 97.5 mm was calculated for each beam, assuming a dose rate of 600 MU/min to dmax, and the ratios between the 6 MV beam and the other beams were calculated, as shown in Table 1. The Varian TrueBeam delivers 600 MU/min for the 6, 10, and 15 MV beams, while the 10 MV FFF beam can operate at 2400 MU/min. Therefore, the actual Cherenkov light yield for the 10 MV FFF beam under clinical conditions can be approximately four times higher than the value presented in Table 1. Considering the clinical deliverable dose rates, the 10 MV FFF beam is expected to produce the largest Cherenkov light yield among all investigated beam energies.

**TABLE 1:** Normalized Cherenkov light yield at a depth of 97.5 mm for each photon beam. The ratios between the 6 MV beam and the other beams, under the condition of 600 MU/min delivery to dmax for all beams, are presented.

Beam type	Cherenkov light yield (photons/Gy)
6 MV	1.00
10 MVFFF	1.41
10 MV	1.73
15 MV	2.08

**FIGURE 8:**
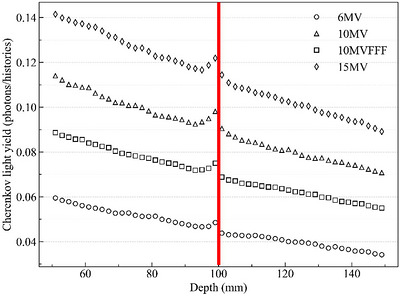
The Cherenkov light yield divided by the number of histories around the thin gold. The scored volume was 20 × 20 × 2 mm^3^. The red line shows the position of the inserted gold.

A localized increase in Cherenkov light yield was observed at approximately 10 cm depth immediately upstream of the gold implant across all beam energies, as shown in Figure 8. The Cherenkov light yield upstream of the gold increased, compared to the Cherenkov light yield without the gold, by 6.12, 7.16, 9.11, and 7.01% for the 6, 10 FFF, 10, and 15 MV beams, respectively. The increase can be attributed to the higher secondary‐electron yield from the high‐atomic‐number gold plate. Additionally, the gold preferentially scattered electrons backward, resulting in localized increases in both the deposited dose and the Cherenkov light yield. Although the 10 MV beam exhibited the highest Cherenkov light yield near the gold implant under identical irradiation conditions, the dose rate significantly affected the observed yield.

### 3.2 Experiment

**FIGURE 9:**
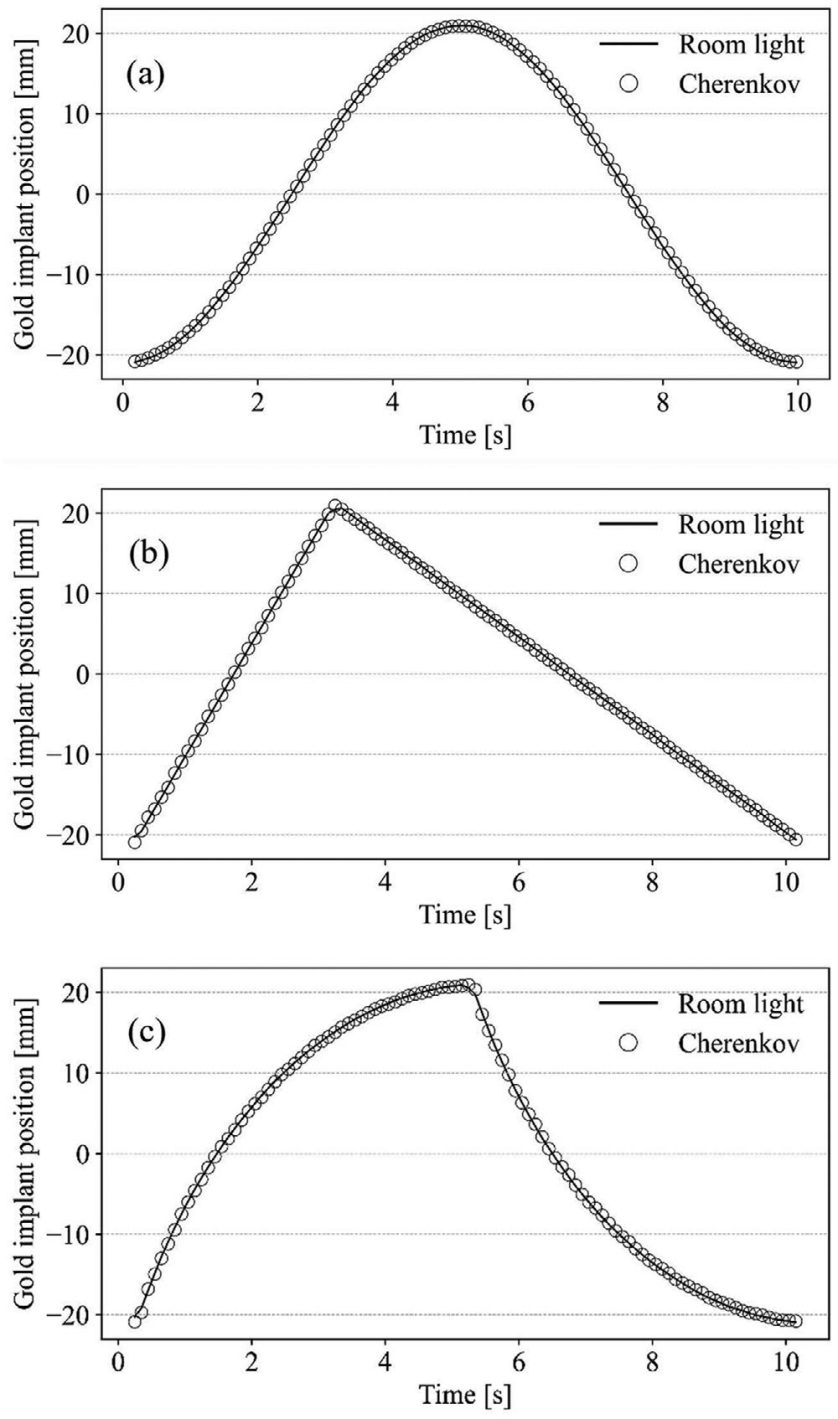
Gold implant position in a bright room and obtained by Cherenkov light. (a): sinusoidal, (b): sawtooth, and (c): sharkfin motion for 10s cycle time.

**FIGURE 10:**
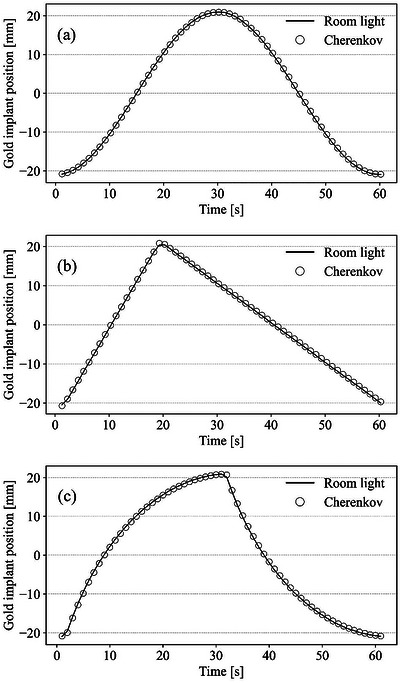
Gold implant position in a bright room and obtained by Cherenkov light. (a): sinusoidal, (b): sawtooth, and (c): sharkfin motion for 60s cycle time.

In Figure 3, we demonstrated that the motion of the gold implant in water is visible in a dark room due to the enhanced Cherenkov emission around it. The positions of the gold implant tracked in both bright and dark room conditions are presented for each programmed motion pattern with 10‐s and 60‐s cycle times in Figures 9 and 10, respectively.

**FIGURE 11:**
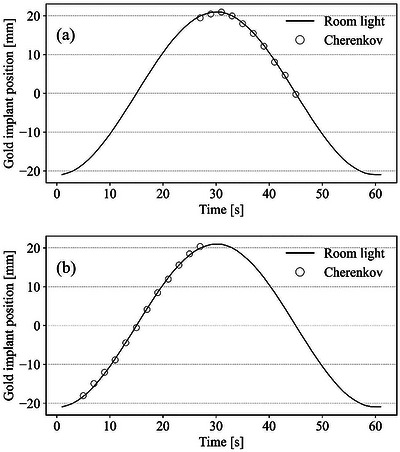
The position of the gold implant with a sinusoidal motion in a bright room and obtained by Cherenkov light for two treatment arcs: (a) is Arc A, and (b) is Arc B.

In the bright room, the calculated repeatability of the marker position was 0 mm at most points, indicating consistent detection under visible light. The mean ± SD repeatability for the 10‐s cycles was 0.04 ± 0.03 mm (sinusoidal), 0.03 ± 0.04 mm (sawtooth), and 0.02 ± 0.02 mm (sharkfin); for the 60‐s cycles, it was 0.02 ± 0.02 mm, 0.03 ± 0.03 mm, and 0.02 ± 0.02 mm, respectively. The mean ± SD accuracy for the 10‐s cycle time, computed as the absolute positional difference between the mean position measured under bright‐room conditions and the position derived from Cherenkov imaging (Equation 2), was 0.09 ± 0.06 mm (sinusoidal), 0.10 ± 0.09 mm (sawtooth), and 0.12 ± 0.12 mm (sharkfin). In contrast, it was 0.13 ± 0.09 mm, 0.17 ± 0.08 mm, and 0.20 ± 0.11 mm for the 60‐s cycles, respectively. These results indicate that the Cherenkov‐derived positions closely match the programmed positions, with differences of less than a sub‐millimeter.

Dynamic imaging of VMAT fields with programmed sinusoidal motion was obtained. The comparison between the gold implant positions acquired in a bright room and those measured using Cherenkov light in a dark room is shown in Figure 11. In this experiment, image acquisition was terminated at the end of treatment; therefore, the recorded images did not span a complete motion cycle. The average accuracy for Arc A and Arc B was 0.19 mm and 0.03 mm, respectively.

## 4 DISCUSSION

To validate the Monte Carlo simulation, the relationship between PDD and Cherenkov light yield was investigated at different photon beam energies. The results showed that the Cherenkov light yield overestimated the PDD, particularly in deeper regions due to beam hardening. These findings were consistent with those of a previous study.^9^ Any differences between our results and those of previous studies may reflect variations in the voxel size used for scoring, the field size, and the phase‐space files employed. Voxel dimensions and scoring volume definitions can bias central‐axis profiles by averaging over high‐gradient regions (e.g., near dmax and in the build‐up/penumbra). Field size and jaw/collimator settings alter the contribution of head scatter, thereby altering spectral hardening with depth and the relative Cherenkov yield. Furthermore, the phase‐space energy spectrum and placement (e.g., scoring plane location and ‘jaws’ placement) affect the incident energy‐angular distributions entering the phantom, which in turn change both the PDD and Cherenkov light yield.

Monte Carlo simulations in this study demonstrated an increase in Cherenkov photon emission near the gold implant for all beam energies. Our simulations suggest that, for Varian TrueBeam photon beams, the 10 MV FFF beam delivered at 2400 MU/min produces approximately 2.7‐fold higher Cherenkov light yield than the 15 MV beam delivered at 600 MU/min, consistent with a previous study.^23^ Based on these findings, the 10 MV FFF beam was selected for our experiments, as its fourfold higher dose rate (MU/min) enabled a stronger Cherenkov signal. The high‐dose‐rate 10 MV FFF beam produced greater Cherenkov light yield within the phantom, thereby improving the visualization and tracking accuracy of the gold implant under both static and dynamic conditions.

The position tracking of the gold implant using Cherenkov light imaging demonstrated excellent agreement with the corresponding positions obtained under bright room conditions, supporting the validity of the tracking approach. The results of VMAT irradiation experiments further confirmed that Cherenkov imaging could accurately track the gold implant's motion in real time, even with the most advanced and sophisticated dose‐delivery technique. Maintaining high tracking accuracy for complex motion patterns is essential for potential radiotherapy applications, where accurate fiducial‐based motion tracking is required to support motion management and precise dose delivery using MV photon beams.

We compared the proposed Cherenkov‐based fiducial tracking technique with representative motion‐tracking technologies used in radiotherapy, including X–ray–based, MR‐guided, electromagnetic/RF transponder, and ultrasound‐based tracking (Table 2). One of the advantages of the current technique over some other technologies is its ability to perform in situ motion tracking during MV delivery at high temporal resolution, without additional imaging dose beyond the treatment beam, and at a lower cost. Additionally, the technique is free of the limitations imposed by EM‐ and ultrasound‐based devices, which require additional equipment to be placed near the patient. The disadvantages include a depth‐dependent detectability limitation caused by optical attenuation in tissue and a restriction to 2D tracking due to the current single‐camera implementation.

**TABLE 2:** Representative motion tracking systems for internal structures/implanted markers in radiotherapy.^33^

System (vendor)	Method	Accuracy	3D?	Ionizing dose?	Key limitation
ExacTrac / Novalis (Brainlab)^27^	kV stereo X‐ray	∼1 mm	Yes	Yes	Intermittent; imaging dose
CyberKnife Synchrony (Accuray)^28^	kV X‐ray + surrogate	< 1 mm	Yes	Yes	Imaging dose; model‐dependent
MR‐linac^29^	cine MRI	∼1 mm	Yes	No	High cost/complexity
Calypso (Varian)^30^	EM beacons	< 1 mm	Yes	No	Requires implants
RayPilot (Micropos)^31^	EM transponder	>1 mm	Yes	No	Invasive placement
Clarity Autoscan (Elekta)^32^	Ultrasound	< 1 mm	Yes^*^	No	Probe/setup dependence
This study	Cherenkov light	< 1 mm	No	No	Optical depth; 2D only

^*^3D is implementation/workflow dependent.

Despite these promising results, several limitations of the proposed motion‐tracking technique should be addressed in future studies. One of the primary challenges is the inherently low Cherenkov photon yield, which led us use the 10 MV FFF beam, as it provides the highest available dose rate of 2400 MU/min on the Varian TrueBeam system. Monte Carlo simulations indicated that the 15 MV beam could generate more Cherenkov photons per incident photon near the gold implant. However, the 15 MV beam is limited to a maximum dose rate of 600 MU/min on the Varian TrueBeam system, which was insufficient to capture clear implant motion images without a more sensitive camera. Optimization of the camera setup, including camera sensitivity, exposure time, aperture/gain settings, and the distance between the gold implant and the camera, will likely further improve the Cherenkov signal‐to‐noise ratio.

Another limitation of the current study is the use of water as the experimental medium, rather than human tissue or a radiologically tissue‐equivalent material. In the visible range, Cherenkov photons attenuate more strongly in biological tissue than in water; consequently, a clinically measurable signal for marker visualization is expected to be substantially reduced. This behavior reflects the well‐documented wavelength dependence of tissue scattering and absorption, which has been observed explicitly in Cherenkov imaging of patients.^24^, ^25^, ^26^ This limitation could be mitigated by using a more sensitive camera capable of detecting lower‐intensity optical signals. Another approach is to shift detection toward longer wavelengths, such as the near‐infrared (NIR), where tissue attenuation is generally lower, using a specialized camera.^11^ Several studies have shown that converting blue‐weighted Cherenkov emission into red/NIR bands via fluorophores or by detecting near‐infrared fluorescence can improve depth sensitivity during radiotherapy.^12^, ^13^

Nevertheless, achievable NIR penetration remains highly context‐dependent and varies with irradiance (power density), wavelength, and tissue optical properties; a review of NIR photon penetration highlights these limitations, particularly for intracranial applications.^14^ Collectively, these considerations suggest that translating water‐tank performance to the clinic will require sensitivity‐optimized optics, careful wavelength selection, and potentially fluorophore‐mediated spectral shifting or model‐based attenuation correction to account for patient‐specific tissue optics.^25^ Importantly, the marker implant depth of approximately 10 cm in water should not be interpreted as a clinically achievable optical tracking depth in tissue. Water provides an optically favorable environment with substantially lower scattering and absorption than biological tissue in the visible range; therefore, the present experiment represents a best‐case validation of the tracking methodology. With the current visible‐wavelength detection and camera configuration, initial clinical applications are more likely to be limited to superficial targets (near‐surface sites), unless sensitivity‐optimized optics and/or longer‐wavelength detection approaches are implemented and validated in tissue‐equivalent media.

Furthermore, the motion of the gold implant occurs in three‐dimensional (3D) space, yet the current experimental setup relies on a single camera, which inherently limits tracking accuracy. To achieve precise 3D motion tracking, multiple strategically positioned cameras can capture motion from different angles around the patient. Optimizing both camera placement and the number of cameras required for robust real‐time monitoring will be essential for the practical clinical implementation of this technique.

Without further advances in optical technology to overcome the inherent limitations of low‐intensity Cherenkov photons, the current technique is limited to dynamic tracking of a gold marker implanted at a shallow depth. Hence, we envision potential application sites for this technology include treatments for tumors near the skin surface, such as breast, extremity, and head and neck cancers.

## 5 CONCLUSIONS

This study demonstrated the potential of Cherenkov light imaging as a precise and reliable method for tracking the motion of gold implants during radiation therapy, using both Monte Carlo simulations and experiments. The simulations showed that the Cherenkov light yield increased around the gold implant due to the production of secondary electrons, and this increase closely correlated with the PDD curves. Furthermore, the relationship between photon beam energy and two quantities (the dose and the Cherenkov light yield) was evident across the Varian TrueBeam photon beams used in this study.

The accuracy of Cherenkov light imaging was assessed by employing static and dynamic experimental setups with 10 MV FFF photon beams. Under static beam conditions, the position of the gold implant determined using Cherenkov light showed excellent agreement with the position measured under bright room conditions for each programmed motion. Additionally, during VMAT irradiation, the Cherenkov light imaging technique successfully tracked the implant's sinusoidal motion with high precision.

These findings have promising implications for the future of image‐guided radiation therapy. The results suggest that Cherenkov light imaging can be further developed to improve tumor localization accuracy. This development could enhance the effectiveness of radiation therapy while potentially reducing radiation exposure to surrounding healthy tissues, without the need for a costly apparatus.

## AUTHOR CONTRIBUTION

Keita Okazaki drafted the manuscript, performed the Monte Carlo simulations and experiments with Yoichi Watanabe, and analyzed the acquired data. Eric Brost assisted with simulation code and provided critical review of the manuscript. Gopishankar Natanasabapathi performed additional experiments to support the data and contributed to the critical review. Yoichi Watanabe provided overall supervision and guidance, led discussions with the co‐authors, reviewed the results, and critically revised the manuscript.

## CONFLICT OF INTEREST STATEMENT

The authors declare no conflicts of interest.

## ETHICS STATEMENT

This study involved phantom measurements and Monte Carlo simulations only and did not include human participants, patient‐identifiable information, or animal experiments. Therefore, institutional review board (IRB) approval and informed consent were not required.

## Data Availability

The data that support the findings of this study are available from the corresponding author upon reasonable request. Restrictions apply to the availability of certain data (e.g., manufacturer‐provided phase‐space files), which were used under license and are not publicly available.
